# Effect of 2-Hydroxy-4-Methoxy Benzoic Acid from the Roots of *Hemidesmus indicus* on Streptozotocin-induced Diabetic Rats

**DOI:** 10.4103/0250-474X.58180

**Published:** 2009

**Authors:** M. Gayathri, K. Kannabiran

**Affiliations:** Biomolecules and Genetics Division, School of Biosciences and Technology, VIT University, Vellore-632 014, India

**Keywords:** 2-Hydroxy-4-methoxy benzoic acid, Ca^2+^ ATPase, *Hemidesmus indicus*, lipid peroxidation

## Abstract

The aim of the present study was to investigate the effect of 2-hydroxy-4-methoxy benzoic acid isolated from the roots of *Hemidesmus indicus* on plasma glucose, plasma, erythrocyte and erythrocyte membrane lipid peroxidation and membrane-bound Ca^2+^ ATPase activity in streptozotocin-induced diabetic rats. In our study, diabetic rats had increased levels of blood glucose and lipid peroxidation in plasma, erythrocytes and erythrocyte membrane and decreased level of plasma insulin and decreased activity of low affinity Ca^2+^ ATPase in erythrocytes. Restoration of plasma insulin and glucose in diabetic rats indicates the effect of HMBA on insulin, glucose and lipid peroxidation. HMBA also restored diabetes-induced alterations in the activity of membrane-bound Ca^2+^ ATPase. Based on the results of this study it can be concluded that HMBA mediated normalization of membrane-bound ATPase in erythrocytes is due to improved glycemic control and antioxidant activity.

*Hemidesmus indicus* (Asclepiadaceae) is one of the indigenous Ayurvedic medicinal plants commonly available and widely distributed throughout India. The root bark of this plant has been used as a traditional medicine in the treatment of biliousness, blood diseases, diarrhea, respiratory disorders, skin diseases, syphilis, fever, bronchitis, asthma, eye diseases, epileptic fits in children and also used to treat kidney and urinary disorders, loss of appetite, burning sensation and rheumatism[1]. *H. indicus* is also employed in traditional medicine for the treatment of gastric ailments[2]. It mainly consists of essential oils and phytosterols such as hemidesmol, hemidesterol and saponins. Isolation of 2-hydroxy-4-methoxy benzoic acid (HMBA) from *H. indicus* was already reported[3]. It was reported that it exists as white needle-shaped crystals, soluble in water, methanol and chloroform and has a melting point of 155–158° and λ_max_ 260 nm[3]. The presence of a benzene ring, methoxy group and hydroxyl group in the structure of HMBA having the molecular formula of C_8_H_8_O_4_ and the molecular weight was calculated as 168[3]. The concentration of HMBA in the root bark of *H. indicu* s was in the range of 0.03–0.54%[4]. It possess potent antiinflammatory, antipyretic and antioxidant properties[5]. It also neutralizes viper-venom-induced changes in serum phosphatase and transaminase activity in male albino rats and also reduced free radical formation[6]. The compound also has an adjuvant effect and antiserum potentiating activity against viper venom[7]. ACGIH, IARC, NIOSH, NTP and OSHA do not list it for carcinogenicity. The protective effect of *H. indicus* against rifampicin-and isoniazid induced hepatotoxicity in rats[8], as well as CCl_4_ and paracetamol-induced hepatic damage[9], is known. Recently we have reported the antidiabetic activity of the aqueous extract of *H. indicus* in streptozotocin-induced diabetic rats[10]. In the present investigation HMBA was isolated from the roots of *H. indicus* and tested for antidiabetic and antiperoxidative activity in STZ-induced diabetic rats.

The root of *H. indicus,* was collected from the Morappur forest area, Dharmapuri District, Tamil Nadu, and authenticated by the forest department, where a voucher specimen (FDSC201) was also submitted. Roots of *H. indicus* was washed with distilled water, shade dried, powdered and stored in an air-tight container until further use. The protocol used for animal experimentation was approved by the Institution Animal Ethical Committee, (IEAC, VIT University).

The methanol root extract (200 g) was mixed with methanol-chloroform (9:1, v/v) and centrifuged at 2000 rpm for 15 min. The silica gel was then added to the suspension and evaporated to dryness. The residue was placed on top of the silica gel (60-120 mesh) column (80x600 mm) packed with petroleum ether. The column was eluted with petroleum ether-benzene (4:1, 2:1, 1:1, 0:1, 2000 ml each), benzene-chloroform (9:1, 4:1, 2:1, 1:1, 0:1, 2000 ml each) and chloroform-ethanol (98:02, 95:05, 90:10, 2000 ml each). The fraction collected were evaporated to dryness and tested for homogeneity using TLC and tested for antidiabetic activity.

The active compound eluted with methanol-chloroform (95-05) was taken for further purification using a silica gel (100-200 mesh) column (30×200 mm). Subsequently eluted in accordance with the polarity of the solvent, petroleum ether-benzene (9:1, 4:1, 2:1, 1:1, 0:1, 500 ml each), benzene-chloroform (2:1, 1:1, 0:1, 500 ml each)[3]. The fractions eluted were evaporated to dryness and tested for homogeneity on TLC, and antidiabetic activity. The active compound eluted with benzene was further crystallized. A mixture of silica gel 60G:60H (1:1, w/w) in water was degassed and poured on to TLC plates (20×10 cm×1 mm). The plates were activated at 110° for 2h, and then stored in a moisture-free cabinet. The plates were developed in a mixture of chloroform-methanol (1:1, v/v) and benzene-chloroform (1:1, v/v). The spots were developed in an iodine chamber and the spot obtained was scrapped and subjected to spectral studies.

The UV spectra of the purified fraction were carried out in a Perkin-Elmer Spectrophotometer (Model 5503) in the wavelength region of 200 to 800 nm. Nuclear magnetic resonance spectra (^13^C and ^1^H NMR) were measured using DMSO as solvent in 200 MHz. Gas chromatography combined with mass spectrum of the purified fraction was recorded using AcqMethod AUTO GCMS. The purified fraction was also identified by X-ray crystallographic analysis. The crystal was mounted on a goniometer head and X-ray data were collected using CuKa radiation. The structure was solved using Direct Method procedure using computer programme, SHELX-86 and refined by SHELXS-93. The Zortep plot of the compound was plotted. The physical characteristics like melting point, boiling point and the solubility of the compound was determined. Based upon the spectroscopic and crystallographic studies, the structure of the active compound was identified. The purity of the isolated compound was confirmed with the standard 2-hydroxy-4-methoxy benzoic acid (HMBA) purchased from the Sigma-Aldrich Chemical Co., USA (Cat # 17,347-9).

Male Wistar rats (150-200g) were purchased from Tamil Nadu Veterinary Animal Science University, Madhawaram, Chennai, and housed under standard husbandry conditions (30±2°, 60-70% relative humidity and 12 h:12 h day-night cycle) and allowed standard pellet rat feed and water *ad libitum.* Animals were housed in standard environmental conditions (as per Institutional Animal Ethical Committee norms). Tolbutamide was purchased from Apollo Pharmacy, Vellore, India.

Diabetes was induced experimentally in rats by a single intraperitoneal injection of freshly prepared solution of streptozotocin (STZ, Sigma, St. Louis, USA) at a dose of 35 mg/kg body weight in 0.1 M citrate buffer, pH 4.5. The STZ-treated animals were considered to be diabetic, if the blood glucose values were above 250 mg/dl and stabilized for a period of 7 days and those animals alone were selected for this study. Animals were divided into four groups of six animals each. Group I served as a control; group II had STZ-induced surviving diabetic rats; group III served as a positive control and received a standard hypoglycemic agent, tolbutamide (100 mg/kg) by oral intubation method and group IV diabetic rats treated with the HMBA (500 μg/kg) for 7 weeks by oral intubation method. Blood samples were collected from the tail vein using aseptic precautions at the end of the treatment period in the tube containing potassium oxalate and sodium fluoride for the estimation of plasma glucose by glucose oxidase method[11], and plasma insulin (Radioimmunoassay kit, Pharmacia, Sweden)[12]. The levels of TBARS in plasma[13], erythrocytes, erythrocyte membrane and erythrocyte membrane Ca^2+^-ATPase activity[14] were measured.

Statistical analysis was performed using SPSS software package, version 9.05. The values were analyzed by one way analysis of variance (ANOVA) followed by Duncan's multiple range test (DMRT). All the results were expressed as mean ±SD for six rats in each group *P*<0.001 were considered as statistically significant.

The FTIR (KBr) spectrum showed bands at 1650 and 1620 cm^−1^ for COOH grouping and aromatic moieties. The ^13^C NMR spectrum of the compound exhibited signals for eight carbons, of which a quartlet at δ174.2 ppm supported the presence of a COOH group. The remaining three doublets at δ 101.0, 108.1 and 132.4 ppm and three singlets at δ164.6 and 168.8 ppm indicated the presence of a trisubsituted aromatic ring in the molecule. ^1^H NMR (CDCI_3_) spectrum displayed, besides the signals for OMe (δ3.86 ppm) and intramolecular hydrogen bonded OH protons (δ 10.59 ppm), two ortho coupled signals at δ 6.51 and 7.86 ppm (J=9 Hz), the former being split further by a meta related proton (with J=2Hz), which in turn resonated as a doublet at δ 6.45 ppm (J=2Hz). The spectral data of the compound showed the m/z at 168, corresponding to the molecular weight and the molecular formula C_8_H_8_O_4_. Based on the spectral data the pure compound was identified as HMBA and the structure was assigned for the compound. The X-ray crystallographic data also confirmed that the compound is HMBA.

The % yield of HMBA obtained was about 2.5% and the purity was 99%. The physical characteristic features of HMBA are given Table 1. The effect of HMBA on plasma glucose, insulin and erythrocyte membrane low affinity Ca^2+^-ATPase activity is given in Table 2. Oral administration of aqueous solution of HMBA significantly (*F*>0.05; *P*<0.001) reduced the blood glucose level and increased the level of plasma insulin to near normal level when compared to untreated control rats. In diabetic rats the activity of low affinity Ca^2+^-ATPase was considerably decreased. Oral administration of HMBA increased the activity of low affinity Ca^2+^-ATPase significantly (*F*>0.05; *P*<0.001) to normal level. The effect of the HMBA on plasma, erythrocytes membrane and erythrocytes lipid peroxidation is given in Table 3. The significantly (*F*>0.05; *P<*0.001) elevated levels of TBARS in plasma, erythrocytes and erythrocyte membrane of diabetic rats were reduced significantly (*F*>0.05; *P<*0.001) to near-normal levels upon treatment with HMBA.

**TABLE 1:** MAJOR PHYSICAL CHARACTERISTICS OF HMBA

Physical Properties	Characteristics
Solubility	Water, methanol, chloroform, benzene
Shape	White shiny crystals
Melting Point	155-158°
UV (λ max nm)	260
IR (cm-1)	1500-1600 (unsaturated acid group)
^1^H-NMR	Benzene ring with one hydroxyl and one methoxy group
^13^C-NMR	Trisubsituted aromatic and COOH group
Mass Spectrometry (mol wt)	168

**TABLE 2:** EFFECT OF HMBA ON PLASMA GLUCOSE, INSULIN LEVELS AND ERYTHROCYTE MEMBRANE CA^2+^-ATPASE ACTIVITY

Groups	Dose (mg/kg bw/day)	Plasma glucose (mg/dL)	Plasma insulin (μU/ml)	Ca^2+^-ATP ase activity (μ mole Pi/mg protein/h)
Normal	-	69±3.19	15.62±1.3	0.30±0.12
Diabetic control	-	288±3.28	6.89±1.0	0.10±0.05
Diabetic + Tolbutamide	100	197±2.98*	13.90±1.4*	0.2±0.03*
Diabetic + HMBA	0.5	75±2.0*	16.0±1.3*	0.32±0.02*

Each value is mean±SD for six rats in each group.

* Values are statistically significant when compared to diabetic control at F>0.05(ANOVA) and *P*< 0.001 (DMRT).

**TABLE 3:** EFFECT OF HMBA ON PLASMA, ERYTHROCYTE AND ERYTHROCYTE MEMBRANE TBARS LEVELS

Groups	Dose (mg/kg /day)	TBARS		
		
		Plasma (nmol/ml)	Erythrocyte membrane (nmol/mg protein)	Erythrocytes (pmol/mg Hb)
Normal	-	1.6±0.12	0.55±0.09	0.75±0.07
Diabetic control	-	3.9±0.05	0.88±0.87	0.9±0.05
Diabetic+tolbutamide	100	2.5±0.03*	0.81±0.27*	0.88±0.04*
Diabetic+HMBA	0.5	1.4±0.02*	0.58±0.95*	0.65±0.04*

Each value is mean±SD for six rats in each group.

* Values are statistically significant when compared to diabetic control at F>0.05(ANOVA) and *P*< 0.001(DMRT).

In our study, diabetic rats had elevated level of blood glucose and treatment with HMBA and tolbutamide reduced blood glucose level significantly, which could be associated with increased secretion of insulin. For induction of diabetes low concentration of STZ (35 mg/kg) was used and diabetic rats still possess functional islets cells. It was well documented that during diabetes mellitus reactive oxygen species (ROS) were generated and the increased levels of ROS can cause increased lipid peroxidation and there by elevation of lipid peroxidation markers. In the present study, the elevated levels of TBARS in plasma, erythrocyte and erythrocyte membrane are consistent with earlier reports[15]. Treatment with HMBA and tolbutamide reduced the levels of TBARS to near normal, which could be associated with improved glycemic control. The observed effect of HMBA may be due to its antioxidant activity. Our report is consistent with the earlier reports that HMBA possess significant antioxidant activity[5].

Hyperglycemia can cause glycosylation of proteins and cellular lipid peroxidation, which, in turn, can cause inhibition/reduction in the activity of Ca^2+^-ATPases. This in turn, can affect the intracellular concentration of Ca^2+^, thereby alter the signal transduction pathways, and also affect contractility and excitability and cellular dysfunctions[16]. Diabetic rats had decreased activity of low affinity Ca^2+^-ATPase as a consequence of interaction of glucose with Ca^2+^-ATPase[16]. The activity of low affinity Ca^2+^-ATPase was found to decreased in the erythrocytes of diabetic rats. Increased lipid peroxidation can, in turn, diminish the activity of low affinity Ca^2+^-ATPase in erythrocyte membrane when exposed to a higher glucose concentration-containing medium[16]. Diabetic rats treated with HMBA and tolbutamide showed the reversal of low affinity Ca^2+^-ATPase to near normal level which might be associated with decreased peroxidative damage to erythrocyte membrane phospholipids along with improving glycemic control. Thus HMBA treatment restored low affinity Ca^2+^-ATPase by decreasing lipid peroxidation and also improves glycemic control. Insulin has been reported to have direct effect on regulation of the membrane bound (Ca^2+^+Mg^2+^)-ATPase[17]. Low-affinity Ca^2+^-ATPase is considered to be responsible for the shape and deformability of the erythrocyte membranes[18]. In our study, diabetic rats showed decreased activity of low affinity Ca^2+^-ATPase and this could be due to insulin deficiency and insulin being the regulator of the enzyme. Treatment with HMAB restored the level of low affinity Ca^2+^-ATPase, which might be associated with insulin secretary effect. The results of this study demonstrate that HMBA exhibits promising antidiabetic activity and also helps to maintain glycemic control by curbing ROS.
